# Drug repurposing in cardiology

**DOI:** 10.1007/s12471-020-01430-0

**Published:** 2020-05-06

**Authors:** B. O. van Driel, J. van der Velden

**Affiliations:** Department of Physiology, Amsterdam Cardiovascular Sciences, Amsterdam UMC, Vrije Universiteit Amsterdam, Amsterdam, The Netherlands

In this month’s issue of the *Netherlands Heart Journal*, van de Bovenkamp et al. [1] describe their study design of the DoPING-HFpEF trial, a placebo-controlled cross-over trial that studies the effect of trimetazidine on diastolic function and cardiac energetics in heart failure with preserved ejection fraction (HFpEF). It seems that in the absence of specific therapies for HFpEF, cardiologists might have to resort to using doping substances to treat their patients. Trimetazidine was added to the World Anti-Doping Agency (WADA) Prohibited List on 1 January 2014, and 2 years later its analogue meldonium caused turmoil in international sports when it was added to the list of forbidden substances and contributed to the banning of an unprecedented number of top-level sportsmen and women. Both drugs are known for their cardioprotective effects and enhancement of glucose oxidation by limiting beta-oxidation through the inhibition of synthesis or carnitine-facilitated transport of fatty acids.

Conventional heart failure therapies that alter haemodynamics have a limited effect on HFpEF, so instead a therapy is being sought in metabolic modulation: altering the metabolic state of the cardiac cell in order to improve its function. Trimetazidine does not alter haemodynamics; instead it provides the heart muscle with more energy (adenosine triphosphate, ATP) per molecule of oxygen. Subsequently the heart is provided with more energy for the same amount of heart muscle perfusion. The idea is not new, since trimetazidine is used as a therapy for angina pectoris and was marketed in the 1970s, around the same time as atenolol, the haemodynamic-altering therapy for angina pectoris. Metabolic modulation is also a topic of interest in hypertrophic cardiomyopathy (HCM). A trial with perhexiline, which limits beta-oxidation by the inhibition of carnitine palmitoyltransferase 1, showed that it corrects energy deficiency and diastolic dysfunction in advanced HCM [2]. Trials with ranolazine [3], a late sodium current inhibitor, and trimetazidine [4] in non-obstructive HCM showed no effect on exercise capacity and diastolic function despite promising preclinical data [5]. The effect of trimetazidine on myocardial energy efficiency in genotype-positive, phenotype-negative HCM mutation carriers is currently being investigated in the ENERGY trial [6]. Treatment success may depend on the disease stage and corresponding alterations in metabolism as a result of progressive cardiac remodelling. Therefore it is essential to carefully characterise the study population and use advanced clinical imaging to measure the effect of metabolic modulators.

Now, for the first time, the DoPING-HFpEF trial will use trimetazidine in HFpEF to research if supplying the energy-deprived heart muscle with extra energy will improve its function. Van den Bovenkamp and colleagues have carefully characterised their HFpEF study population and will measure pulmonary capillary wedge pressure during exercise by right heart catherisation, as well as cardiac energetics by determination of the phosphocreatine/ATP ratio with phosphorus-31 magnetic resonance spectroscopy. It is a prime example of translational medicine and drug repurposing: knowledge acquired in preclinical research being applied directly in a clinical setting with an approved drug.

In these challenging times caused by the COVID-19 pandemic, research might be delayed for many months to come. We wish everyone health, strength and lots of energy.
